# *GoFish:* A low-cost, open-source platform for closed-loop behavioural experiments on fish

**DOI:** 10.3758/s13428-022-02049-2

**Published:** 2023-01-09

**Authors:** Victor Ajuwon, Bruno F. Cruz, Paulo Carriço, Champalimaud Research Scientific Hardware Platform, Alex Kacelnik, Tiago Monteiro

**Affiliations:** 1 Department of Biology, University of Oxford, Oxford, UK; 2 Champalimaud Neuroscience Programme, Champalimaud Foundation, Lisbon, Portugal; 3 Present Address: NeuroGEARS Ltd., London, UK; 4 Champalimaud Research Scientific Hardware Platform, Champalimaud Foundation, Lisbon, Portugal; 5 Champalimaud Foundation, Lisbon, Portugal; 6 Domestication Lab, Konrad Lorenz Institute of Ethology, Department of Interdisciplinary Life Sciences, University of Veterinary Medicine Vienna, Vienna, Austria

**Keywords:** Automatic feeder, *Bonsai*, Fish behaviour, Goldfish, Integrated fish conditioning platform, Reversal learning

## Abstract

Fish are the most species-rich vertebrate group, displaying vast ecological, anatomical and behavioural diversity, and therefore are of major interest for the study of behaviour and its evolution. However, with respect to other vertebrates, fish are relatively underrepresented in psychological and cognitive research. A greater availability of easily accessible, flexible, open-source experimental platforms that facilitate the automation of task control and data acquisition may help to reduce this bias and improve the scalability and refinement of behavioural experiments in a range of different fish species. Here we present *GoFish,* a fully automated platform for behavioural experiments in aquatic species. *GoFish* includes real-time video tracking of subjects, presentation of stimuli in a computer screen, an automatic feeder device, and closed-loop control of task contingencies and data acquisition. The design and software components of the platform are freely available, while the hardware is open-source and relatively inexpensive. The control software, *Bonsai*, is designed to facilitate rapid development of task workflows and is supported by a growing community of users. As an illustration and test of its use, we present the results of two experiments on discrimination learning, reversal, and choice in goldfish (*Carassius auratus)*. *GoFish* facilitates the automation of high-throughput protocols and the acquisition of rich behavioural data. Our platform has the potential to become a widely used tool that facilitates complex behavioural experiments in aquatic species.

**Supplementary Information:**

The online version contains supplementary material available at 10.3758/s13428-022-02049-2.

## Introduction

A common framework for the study of animal behaviour and cognition involves presenting stimuli and manipulanda, measuring animals’ movements and responses, and programming outcomes according to selected contingencies, while recording all of this information, including fine temporal details. These efforts have led to the development and use of conventional experimental platforms that satisfy these needs while ensuring replicability across laboratories. Perhaps the archetype of such a system is the Skinner box, originally designed for pigeons and rodents, which uses manipulanda suitable for those taxa, detecting behaviour by the closing of circuits through key-pecking, lever-pressing, or interruption of light beams. Such systems promote and enhance reproducibility, a critical need in contemporary behavioural research. However, studying the behaviour of organisms living under water, such as fish, cephalopods, and crustaceans, poses different technical challenges from those in terrestrial species.

Unlike most behavioural laboratory-based experiments involving mammals and birds, the display of stimuli and delivery of food reinforcers for fish is frequently manually executed by an experimenter, increasing temporal variability and vulnerability to observer effects, while restricting scalability (e.g., Potrich et al., 2022; Schluessel et al., 2022). Similarly, data are often recorded by video but annotated visually or digitised at a later time instead of being processed in real-time, which allows behaviour to control reward through pre-programmed contingencies.

In spite of their huge ecological, neuroanatomical and behavioural diversity, historically fish have been underrepresented in the cognitive and psychological literatures for decades (Bitterman, 2006; Newport, 2021; Shettleworth, 2009), with primates, corvids, pigeons, rodents and more recently dogs being favoured models (though see Bitterman, 1975; Chase & Hill, 1999; Gerlai, 2014; Pouca & Brown, 2017; Salena et al., 2021). Increasing the availability of user-friendly, open-source, experimental platforms that allow for automated testing and data acquisition in fish may help to mitigate this bias (Brock et al., 2017; Gatto et al., 2021).

Advances in zebrafish research highlight how novel technology, increased experimental automation (Aoki et al., 2015; Brock et al., 2017; Guilbeault et al., 2021; Kuroda et al., 2017; Manabe et al., 2013; Miletto Petrazzini et al., 2020; Mueller & Neuhauss, 2012; Santacà et al., 2021; Stewart et al., 2015), and an ideal model fish species, together can help to unravel the links between genes, neural activity, behaviour and cognition (for reviews see Gerlai, 2014, 2020; Kalueff et al., 2013; Meshalkina et al., 2017; Orger & de Polavieja, 2017), with translational impacts for the treatment of disease (Kalueff et al., 2014). Nonetheless, despite the many advantages of zebrafish as models (e.g., their rapid life-cycle, optical transparency enabling non-invasive whole-brain electrophysiological recordings, and a variety of sophisticated genetic tools) the use of zebrafish for more cognitively demanding tasks may be limited (Blaser & Vira, 2014; but see: Gerlai, 2017; Santacà et al., 2021; Sridhar et al., 2021).

Recent attempts (i.e., < 10 years) to improve the automation of behavioural experiments in other fish species using closed-loop systems have shown promising results. For example, Wallace et al. (2020) investigated sex differences in numerical discrimination abilities in mosquitofish using an automated setup that facilitated a range of cognitive tests. Furthermore, automated systems that were originally developed for conditioning experiments in zebrafish (Gatto, Lucon-Xiccato, et al., 2020a; Kuroda et al., 2017; Manabe et al., 2013) have been co-opted for use in guppies (Gatto et al., 2021; Gatto, Testolin, et al., 2020b; Lucon-Xiccato et al., 2018).

However, most of these systems are either commercial solutions and therefore not easily adaptable or accessible (owing to higher costs), or are open-source but require a considerable degree of expertise to operate and adapt, thus lacking the flexibility to be easily applied to multiple experimental situations and/or other subject species.

To address this, we developed *GoFish*, an open-source and expandable platform for dynamic, fully automated behavioural experiments on fish or other aquatic organisms. Our aim with *GoFish* is to provide a platform facilitating high-throughput and highly reproducible research that is (i) open-source, (ii) relatively inexpensive and simple to assemble, (iii) readily modifiable, (iv) supported by a growing community of users, and (v) capable of providing a range of behavioural metrics.

Our platform is inspired by present-day behavioural, cognitive and neuroscience experiments that rely on open-source, community based, *DIY*-type solutions for running and developing new experimental paradigms, as well as for processing and analysing the resulting data streams (Akam et al., 2022; Aoki et al., 2015; Bishop et al., 2022; Buscher et al., 2020; Devarakonda et al., 2016; Geissmann et al., 2017; Guilbeault et al., 2021; Gurley, 2019; Kane et al., 2020; Kapanaiah et al., 2021; Lopes et al., 2021; Mathis et al., 2018; Oh et al., 2017; O’Leary et al., 2018; Pineño, 2014; Siegle et al., 2017; Štih et al., 2019; Swanson et al., 2021; Walter & Couzin, 2021).

Briefly, our system allows for the display of stimuli on a computer screen placed outside (but adjacent to) a tank, the tracking and detection of the subject’s location in real-time through an overhanging camera, the programming of contingencies between fish movements and the delivery of food rewards, and the automatic recording of data in analysable format. Here, we describe the system and present two closed-loop experiments aimed at demonstrating its performance as a research tool. Although we describe an implementation for goldfish, *GoFish* can, in principle, be used with other aquatic species with minimal modifications.

As a proof-of-concept, and inspired by classical experiments (Bitterman, 1975; Engelhardt et al., 1973), we ran two closed-loop discrimination experiments using real-time video tracking. We show that individual goldfish can be trained to (i) associate a signalled location with food reward and reverse preference appropriately when the contingencies are reversed (*Experiment* 1), and (ii) discriminate coloured visual stimuli that switch location between trials (*Experiment* 2).

## Methods

### The *GoFish* platform

**Fig. 1:**
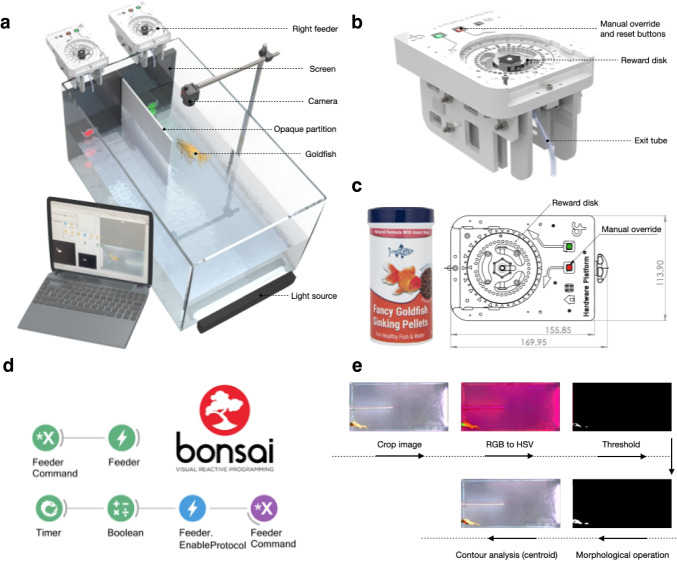
*GoFish* apparatus, pellet dispenser control and specifications, and video tracking pipeline. **a.** 3D view of closed-loop operant chamber. Setup includes two custom-made pellet dispensers, computer screen, USB camera, and light source. **b.** 3D depiction of the pellet dispenser. **c.** Food rewards (*left*) and detailed top view of feeder reward container disk. **d.** Example *Bonsai* workflow for custom pellet dispenser control. The code implements periodic delivery of food pellets. **e.** Real-time video analysis pipeline, see also Supplementary Videos **1** and 2

The setup as presently implemented (Fig. 1) comprises a rectangular prismatic experimental tank (60 x 30 x 36 cm (*length* x *width* x *height*), Table 1) with a 17” LCD computer screen (1920 x 1080; 60 Hz) for stimulus presentation (Table 1), placed directly adjacent to the side of the tank where reward pellets are delivered, (Fig. 1a).

**Table 1:** Parts list

Component Name	Supplier	Reference code	Quantity	Unit price	Total Price
*Clearseal All Glass Aquaruim (24×15×12in; L×H×W)*	Goldfish Bowl, Oxford, UK	N/A	1	£55	£55
*WIFIGDS 17 Inch Monitor*	Amazon	BO8J7JJD3G	1	£81	£81
*1080P 2MP ELP Web Camera*	ELP	ELP-USBFHD08S-UK	1	£72	£72
*Pellet dispenser*	Champalimaud Hardware Platform	FF_GF_SP_V1.1	2	£142	£284
*IREENUO Aquarium Light (11inch)*	*IREENUO*	HDMU_ATL-18-DE	1	£14	£14
*Laptop computer*	Dell	Latitude 5520	1	£537*	£537
*5mm White Acrylic (300×200×5mm)*	Cut My Plastic	N/A	1	£10	£10
*Betta Clear Silicone 310ml*	Goldfish Bowl, Oxford, UK	N/A	1	£13	£13
*Rankie USB 2.0 Extension Cable*	Amazon	B01KWOBK84	3	£5	£15
Combined Total					£1,081

Details of the components used to build one closed-loop behavioural chamber for goldfish learning experiments. Many of the components can be swapped for items of similar functionality to suit particular needs. Pellet dispenser parts list and assembly instructions can be found in a dedicated repository (see Pellet dispenser section for details). Prices are from early 2021, rounded to the nearest pound

*UK educational price (VAT exempt)

Two custom-made, automated pellet dispensers (i.e., feeders (Fig. 1b,c, Table 1) are clamped onto the upper edge of the tank such that pellets fall on the water surface approximately 2 cm from the closest side of the tank, adjacent to the screen.

Each feeder is placed on either side of an opaque, white acrylic divider (fixed with silicone sealant), running perpendicular to the LCD computer screen, 25 cm into the tank. This partition defines the two choice zones of a Y-maze configuration. An overhanging USB camera (1280 x 720 resolution, Table 1) held above the tank records each session (Fig. 1a). A laptop (Table 1) controls task contingencies (stimulus presentation and reward delivery) and video acquisition with a *Bonsai* (Lopes et al., 2015, 2021; Lopes & Monteiro, 2021) custom workflow (see Fig. 1d for an example workflow). A light source (Table 1) is placed outside the tank, opposite to the LCD computer screen (Fig. 1a). The tank is surrounded by opaque Styrofoam panels to visually isolate the fish during experiments. The water level is maintained at approximately 15 cm. In the experiments described below, two identical experimental tanks were run concurrently, with each fish being tested always in the same tank.

### *Bonsai*

The implementation of behavioural tasks and resulting data acquisition is controlled with *Bonsai*. *Bonsai* is a high performance, open-source visual programming software, for which there is an active community of thousands of users (https://github.com/bonsai-rx/bonsai/discussions) and several papers describing its inner workings (e.g., Lopes et al., 2015; Lopes & Monteiro, 2021). *Bonsai* allows users to rapidly develop workflows that can simultaneously manipulate data from various asynchronous input streams (e.g., video, or Arduino controlled pressure sensors), while controlling numerous output devices (e.g., pellet dispensers).

Users can find documentation, video tutorials, online support, and other materials in its accompanying website (BonsaiRX, http://bonsai-rx.org/). Briefly, *Bonsai* workflows are constructed by connecting functions, or ‘operators’ that come in the form of nodes, together (Fig. 1d). These functions are categorised hierarchically within the *Bonsai* Toolbox that appears in the *Bonsai* workflow editor. For example, ‘Source’ functions allow users to easily generate data streams from files or external devices, while ‘Sink’ functions allow users to save data or trigger external outputs (https://bonsai-rx.org/docs/articles/editor.html). These functions can be searched for directly using the *Search* textbox that appears on top of the *Bonsai Toolbox*, saving users the need to search through all of the functions within the *Toolbox* manually. A full list of *Bonsai* functions and their accompanying descriptions can be found here (https://bonsai-rx.org/docs/api/Bonsai.html). To quickly acquaint themselves with the basics of *Bonsai*, users can access common example workflows online (https://bonsai-rx.org/docs/tutorials/acquisition.html), or import them directly into their workflow editor through the *Bonsai Gallery*, which can be accessed via *Tools* in the menu bar of the workflow editor (https://bonsai-rx.org/docs/articles/gallery.html). Example video tutorials on how to quickly implement common workflows for data processing and storage can also be found here: https://bonsai-rx.org/learn/.

### Pellet dispensers

Design and assembly instructions for laser cut acrylic and 3D printed parts for the pellet dispensers are available from the public repository (https://bitbucket.org/fchampalimaud/device.pump.fishfeeder/).

The instructions include PCB manufacturing plans and specifications, as well as downloadable firmware. The dispensers are controlled through *Bonsai* (see example workflow in Fig. 1d).

### Stimuli

**Fig. 2:**
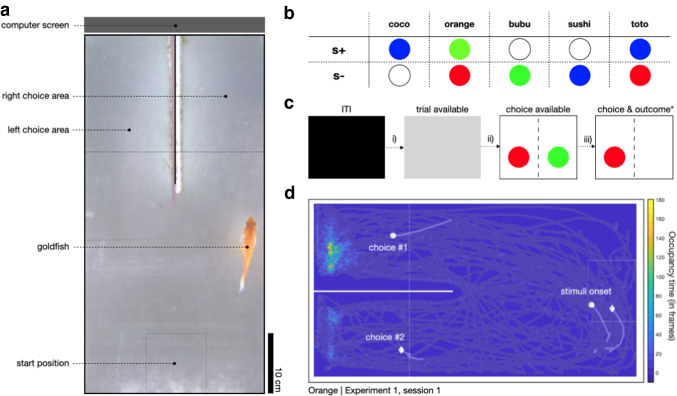
Goldfish were trained to associate colours with food rewards. **a.** Top view of the experimental tank, highlighting the start position and left and right choice areas. **b.** Stimuli colour allocation across subjects. **c.** Trial structure: Every trial started with an ITI, drawn from a uniform distribution (ITI duration, i): min = 20 s, max = 40 s), which was signalled by a black screen. After this a trial became available the screen turned grey, signalling fish could move to the start position. The initiation time ii) was the time between a trial becoming available and a fish entering the start location. As soon as the fish entered the start location, two stimuli would appear on each side of the screen. Choosing S+ led to a pellet reward after a 5-s delay, choosing S- started a new ITI after a 5s delay. The response time iii) was the time between starting a trial and entering the left/right choice areas. **d.** Grey traces show the position tracks for an example animal and session. Markers depict the position of the fish (i.e., centroid) in the second leading to the stimuli onset and choice, for two example trials (shown with different markers), respectively. The underlying heatmap shows a 2D histogram of occupancy times (in number of frames) for the entire session. Same configuration as presented in a., rotated – 90° for presentation purposes

The potential visual stimuli and their positions are only limited by the monitor employed and its chromatic properties and dimensions. For the experiments described here, the main stimuli were coloured circles (red, green, blue and white, 3.5 cm in diameter, Fig. 2b) on a grey background, presented with centres positioned 5 cm from the bottom of the tank and 7 cm from each side wall (Fig. 1a). All stimuli were programmed using custom *Bonsai* (Lopes et al., 2015; Lopes & Monteiro, 2021) and *BonVision code *(Lopes et al., 2021) allowing easy generation and manipulation of visual stimuli. Each fish had a randomly assigned unique pair of colour-reward contingencies (Fig. 2b). We chose colours that have been physiologically (Neumeyer, 1984) and behaviourally (Zerbolio & Royalty, 1983) proven to be discernible by our experimental species (goldfish, *Carassius auratus*). In a pre-experimental, pre-training phase (see details below), we used a white noise rectangle (13.5 x 12 cm, Gaussian: mean = 0, variance = 10) presented on either the left or right arms of the tank, or in both simultaneously, to signal the imminent delivery of reward in early pre-training, or to signal that reward delivery was contingent on fish swimming to a specific location in later pre-training stages.

### Behavioural task control

Task control was fully automated and implemented using a custom *Bonsai* workflow (https://github.com/PTMonteiro/GoFish_Ajuwon_etal_2022). Progress through trials was controlled using real-time video analysis of fish movement. After a variable inter-trial-interval (ITI), fish could advance a trial by swimming into the ‘start zone’. In the main experiment this was a 10 x 10 cm area opposite the rewarded side of the tank that was equidistant to both choice arms (Fig. 2a). Presence in the start zone after the ITI would trigger stimulus presentation, and a subsequent crossing into either the ‘left choice zone’ or ‘right choice zone’ (15 x 15 cm; Fig. 2a) would trigger appropriate contingencies. Outside of these epochs (and locations) the fish position had no influence on the unfolding of the task. Note that the ‘start zone’, ‘left choice zone’ and ‘right choice zone’ were not delineated by physical boundary markings but were defined as specified regions of interest (ROIs) on the video feed corresponding to fixed areas within the experimental tank. Users may wish to make the ROIs visually identifiable to the subjects, as this may influence speed of acquisition. Frames from these ROIs were converted to HSV colour space and a HSV range was applied so as to successfully detect fish. The pixels of the resulting binarized frames from each ROI were summed continuously. Fish entry into the zones was recorded when summed ROI pixels exceeded a set threshold (the value of which was adjusted to each subject prior to the onset of the experiment).

After a session was completed, a timestamped event list was generated as a *.CSV file. The first two columns indicate the event name as a string and a timestamp while the third column contains a number which encodes the particular outcome of events that require extra disambiguating information.

### Fish tracking

To track the fish centroid in real time, a colour thresholding method (Monteiro et al., 2021) was implemented using a custom *Bonsai* workflow. Video was recorded at approximately 33 fps. Frames were cropped down to include only the inside of the tank and converted to HSV colour space. An HSV threshold was applied to isolate the fish body from the overall white background given by the tank’s bottom. Prior to the onset of the experiment, HSV value ranges were manually set for each fish so as to provide robust tracking in spite of individual differences in fish coloration. The resulting binarized region (pixels are either fish or no-fish) was smoothed and the coordinates of the animals’ centroid were extracted.

For each session, a CSV data tracking file is generated which contains the *x* and *y* coordinates of the fish centroid throughout the session in two respective columns. A third column records the luminance of a specified ROI; a central point at the top of the LCD screen on which stimuli are presented to subjects. Recording the luminance of this region provides information about the epoch of the task (Fig. 2c) and therefore allows users to associate fish position with particular epochs of the task, enabling behavioural analysis during trial epochs of interest. For example, a low luminance value indicates an ITI period, an intermediate luminance value indicates the epoch during which a new trial is available and high luminance value represents the post-choice epoch within a trial. Example occupancy data from a representative experimental session, can be found in Fig. 2d.

At the session end, a raw video file of the entire session is also generated, allowing users to perform further tracking analysis offline.

### Subjects

Five goldfish ranging in size between 7 and 10 cm, (age and sex unknown) participated in the current study. Animals were obtained from a local, commercial supplier (Goldfish Bowl, Oxford, UK).

Fish were housed in groups of two or three, in holding aquaria (60 x 35 x 31 cm; (*length* x *width* x *height*)) where they had access to a rock shelter, pebbles and artificial plants. They participated in experiments five times a week on weekdays and fed a total of 24 sinking pellets a day (*Fancy Goldfish Sinking Pellets*, Fig. 1c). This diet was supplemented with spinach following experiments on the last day of the week and bloodworms the day after. Fish were kept under a 12:12 h light:dark cycle using fluorescent lights. Water was maintained at a minimum of 21°C using an internal heater and independent thermometer (pH: 8.2; ammonia: 0 ppm; nitrite: 0 ppm; nitrate: max. 30 ppm). Partial water changes were conducted at the end of each week and internal filters were cleaned every month. Each holding tank was aerated using an air pump.

For each daily session, fish were transported in a plastic jug to its experimental tank and then back to its holding tank at the end of the session. At the start of each day, ~20 L of water from all holding tanks were transferred to the experimental tanks in order to keep the environmental conditions as constant as possible. The experimental tanks were cleaned at the end of each week. All animals had experimental experience with unrelated contingencies.

### Pre-training

Pre-training consisted of three phases lasting a minimum of 18 days in total. Advancing through the phases depended on the individual subject’s performance.

(i) *Experimental tank acclimatisation*
  During a 10-min period, fish were allowed to explore and get acclimated to the tanks, previously baited with 12 food pellets throughout. This phase lasted for 1 day.
(ii) *Choice zone training*
  The aim of this phase was for subjects to learn that swimming into either the left or right choice zone (outside of the ITI) was reinforced. After the ITI (drawn from a uniform distribution: min = 5 s; max = 10 s) during which the screen was black, a white noise rectangle would signal potential food availability in either the left or right choice zones of the tank. Reward was then contingent on fish entering the choice zone signalled with the white noise stimulus. For the first 5 days of this phase, there was one session of 12 trials per day and in the following 5 days, one session of 16 trials per day. Following this, for 3 days fish completed two sessions of 12 trials each per day. Rewards were evenly split across both choice zones and allocated randomly. A session ended either when all trials were completed or after 30 min.
(iii) *Start position training*
  The aim of this phase was for subjects to learn that a start position had to be entered before subsequent behaviour could be reinforced. After the ITI (drawn from a uniform distribution: min = 20 s; max = 40 s), trial availability was signalled by a grey screen. During this period, fish were required to swim first to the back half of the experimental tank into a ‘start zone’ (i.e., > 30 cm, away from the monitor and feeders) to trigger the onset of the white noise stimulus signalling food availability in either the left or right arm. As in the previous phase, reward was then contingent on fish entering a choice zone signalled with the white noise stimulus. This lasted for a minimum of 3 days. Following this, the start zone length was reduced in half (minimum 3 days), and finally to a 10 x 10 cm centred square (minimum 5 days) that was used in the main experiments (Fig. 2a). There were two sessions of 12 trials each per day. To advance through this phase, animals had to successfully consume the 12 food pellets within a 1-h limit in each daily session. Failure to do so would terminate the training session, with fish returned to their holding tanks. The remaining food pellets would be made available by the end of the day in the holding tanks.

### Experiment 1: Acquisition and reversal of a spatial conditioning

#### Acquisition phase

Each fish was presented with one daily session of 24 trials. A trial started with an ITI (drawn from a uniform distribution: min = 20 s; max = 40 s) where the screen was black, and behaviour had no consequences. The ITI offset was signalled by a grey screen (Fig. 2c) and from this moment on, entering the start position (Fig. 2a,d) would trigger the presentation of both visual stimuli (i.e., S+ and S-, see *Stimuli* above) at fixed left/right locations (Fig. 2b,c, counterbalanced across subjects). Fish made choices by entering one of the two choice zones (Fig. 2a). Choosing the S+ side resulted in the delivery of a food pellet after a 5-s delay and the onset of an ITI. Conversely choosing the S-side would start a new ITI after a 5-s delay (Fig. 2c). This experimental phase lasted for 5 days.

#### Reversal phase

This phase followed the same contingencies as acquisition, except that the rewarded side for each animal (and accompanying stimuli location) was swapped, remaining the same after that. This phase lasted for 7 days.

### Experiment 2: Colour discrimination

In this experiment the rewarded side (and S+/S- stimuli) was randomised on a trial-by-trial basis. To make more correct choices the fish had to follow the S+ and S- signals, rather than acquiring a side preference and reversing it. This experiment lasted for 25 days.

### Data analysis

Real-time video tracking (see *Behavioural task control*) was used to control task contingencies and also generated a timestamped event list for each session. Preference and movement time (i.e., initiation and response times) data (Data file 1) were derived from these event lists and analysed using custom Matlab code (R2020a, MathWorks) available at https://github.com/PTMonteiro/GoFish_Ajuwon_etal_2022. Statistical analyses were conducted in RStudio (v1.2.5033; *The R Project for Statistical Computing*, 2018). For statistical analyses, choice proportion data was arcsine square-root transformed to normalise the residuals. One sample, one-sided *t* tests against 50% were used to assess performance at group level.

In both experiments, repeated measures ANOVAs were conducted to assess the effect of session (to detect learning effects). In *Experiment* 2, repeated measures ANOVAs were also conducted to assess the effect of session terciles on trial initiation times and choices (to detect within session satiation or warming up effects). A type-1 error rate of 0.05 was adopted for all statistical comparisons.

### Ethics statement

All experiments were conducted at the John Krebs Field Station and approved by the Department of Zoology Ethical Committee, University of Oxford (Ref. No. APA/1/5/ZOO/NASPA/Ajuwon/Goldfish), and were carried out in accordance with the current laws of the United Kingdom. Animals were cared for in accordance with the University of Oxford’s “gold standard” animal care guidelines. All experimental methods were non-invasive. No food restriction was necessary as fish were fed highly palatable pellets during daily experimental sessions, supplemented by the end of the day in case fish did not eat the minimum daily requirements, and with raw spinach at the end of the last weekly experimental session. Their diet also included blood worms on weekends. Maintenance and experimental protocols adhered to the Guidelines for the Use of Animals in Research from the Association for the Study of Animal Behaviour/Animal Behavior Society (“Guidelines for the Treatment of Animals in Behavioural Research and Teaching,” 2006). On completion, the fish were reintroduced into holding tanks and eventually returned to the supplier.

## Results and discussion

We illustrate the potential of *GoFish* for use in automated, closed-loop behavioural experiments with two discrimination learning experiments with goldfish.

**Fig. 3:**
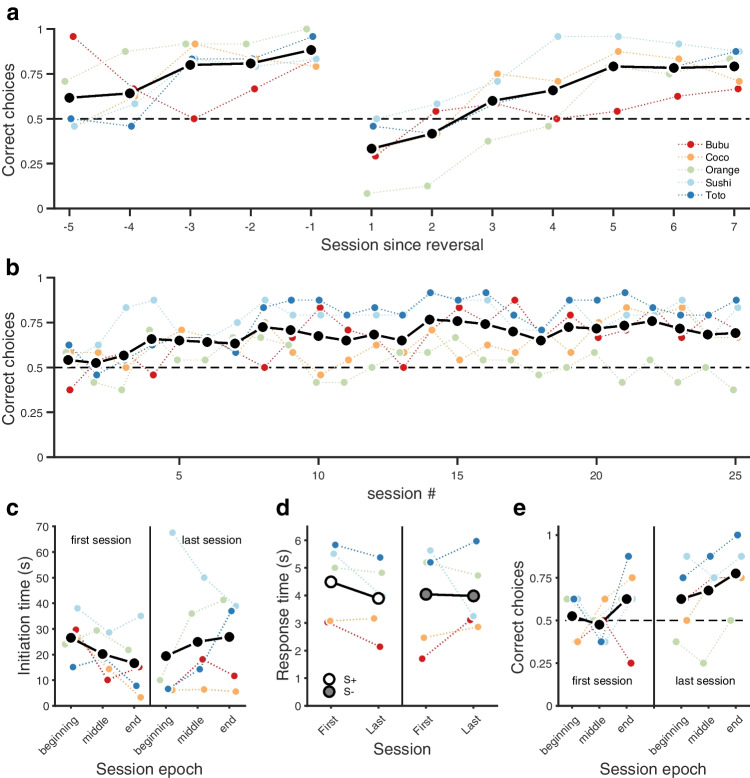
Goldfish learned a colour discrimination task with changing reward/cue/location requirements. **a.** Mean proportion of correct responses for *Experiment* 1 during acquisition and reversal of a spatial discrimination. **b**. Mean proportion of correct responses during the visual discrimination task in *Experiment* 2. **c.** Initiation times for the first and last sessions of *Experiment* 2, split into session terciles. **d.** Response times towards S+ (*left*) and S- (*right*) stimuli for the first and last sessions of *Experiment* 2, split into the first and last portions of the session, respectively. **e.** Proportion of choices as a function of session epoch (*terciles*), for the first (*left*) and last (*right*) sessions of *Experiment* 2, respectively. In all panels, *black* (*white* and *grey*, for panel **d**.) markers show group means and coloured markers the mean proportion of correct responses or median initiation or response times, respectively

In *Experiment* 1, fish (i) controlled the flow of trials by swimming to a start location, which triggered the onset of visual stimuli in two target sites, and (ii) expressed a choice by swimming to either target ROI, which triggered (or not, depending on choice) a food reward, followed by an intertrial interval, at the end of which the ‘start’ ROI became receptive and a new trial could be started. Multiple-trial sessions took place without intervention of the experimenter. This protocol was used in an acquisition and a reversal phase. The experiment and its results (smooth, significant acquisition and reversal, Fig. 3a) are similar to those carried out by Kuroda et al. in zebrafish (*Danio rerio*; Kuroda et al., 2017). Repeated measures ANOVAs with session as the independent variable confirmed a significant increase in preference for the rewarded side in both the acquisition (F_4,16_ = 3.02, *P* < 0.05), and reversal (F_6,24_ = 13.84, *P* < 0.0001) phases. In the last session of each phase, subjects’ preference for the rewarded side was significantly above 50% (acquisition phase: 88% ± 0.04 (mean ± s.e.m.); one-sample t_4_ = 5.41 *P* < 0.01, reversal phase: 79% ± 0.04; one-sample t_4_ = 5.97 *P* < 0.01).

In *Experiment* 2, reward location was randomised on a *trial-by-trial* basis so that the visual coloured stimuli and the spatial cues were no longer redundant, instead only the former were reliable signals for reward. At group level, fish readily learned to track the location of reward (Fig. 3b). A repeated measures ANOVA with session as the independent variable confirmed a significant increase in preference for the side displaying the S+ stimulus (F_24,96_ = 2.01, *P* < 0.01). Data from the terminal session show that the average proportion of rewarded choices was 69% ± 0.09. This result was significantly above 50% (one-sample t_4_ = 2.18, *P* < 0.05) even though one of the five fish failed to learn, as shown in Fig. 3b. Since the same subjects were used in both experiments, carry-over effects from *Experiment* 1 (where reward site was constant across) may have influenced acquisition of the random alternation protocol in *Experiment* 2.

In addition to choice data, we used a real-time tracking pipeline for automated detection and recording of fish entry into the regions of interest (start zone, left choice zone, right choice zone). The tracking data gives direct access to relevant behavioural metrics, such as trial initiation time (i.e., the time animals took to be detected in the start zone following ITI offset; Fig. 2c - ii) and choice response times (i.e., the time from starting a trial to entering one of the choice zones; Fig. 2c - iii).

As a metric for learning and motivational changes, we compared initiation times between the first and last sessions of *Experiment* 2 (Fig. 3c), but found no significant differences (paired t_4_ = – 0.86, *P* = 0.44).

In addition to choice proportion, we measured choice response times. This variable can be extremely informative: in previous studies and protocols it has been found that response times on both single-option and choice trials can be at least as informative regarding preferences and choice mechanisms as choice proportions (e.g., Monteiro et al., 2020). Overall, we found no significant differences in response time between trials in which fish chose correctly or incorrectly (Fig. 3d, first session: paired t_4_ = 1.61, *P* = 0.18, last session: paired t_4_ = 0.29, *P* = 0.44).

Finally, we explored whether the proportion of correct choices varied within sessions by checking for trends across terciles of sessions. Such effects can occur if there are ‘warming-up’ or satiation effects. Once again, we found no significant effects either early or late in training as revealed by repeated measures ANOVAs with session tercile as the independent variable (Fig. 3e, first session: F_2,9_ = 2.29, *P* = 0.16, last session: F_2,9_ = 0.096, *P* = 0.91).

In summary, as a *proof-of-concept* demonstration for *GoFish*, a fully automated, closed-loop, and open-source experimental platform, we show that goldfish can reliably learn to (i) self-initiate trials, (ii) associate a fixed location with reward (iii) reverse their preference when the rewarded location changes, and (iv) associate colours with reward contingencies. We also present temporal data because, although no significant effects were found in this sample study, they illustrate what can be measured and suggest strategies for analysis.

## General discussion

*GoFish* is a new platform for dynamic, fully automated behavioural experiments that facilitates high-throughput, highly reproducible research in fish or other aquatic organisms. *GoFish* is open-source, inexpensive, highly adaptable, and should be supported by a growing community of *Bonsai* users.

Critical to *GoFish*’s functionality is a novel reward pellet dispenser for which we provide design and assembly instructions, and *Bonsai*, the open-source programming language that is used to automate task contingencies and record data.

Using *Bonsai* in *GoFish* improves the user-friendliness of the system compared to proprietary experimental platforms for a number of reasons. *Bonsai* is free and compatible with a vast range of hardware devices meaning that users can easily source components cheaply or use already existing ones. Critically, *Bonsai* is a visual programming language, meaning that users with little or no previous coding experience can quickly develop effective workflows for task control and data analysis. In order to adapt our workflow for different protocols, users will need to learn the basics of *Bonsai*, which can be done through the extensive documentation that exists on the *Bonsai* website, including example workflows and video tutorials.

As a generic experimental platform, *GoFish* provides advances and improvements over more common experimenter-controlled setups currently used in research on fish behaviour and cognition. These improvements have benefits in four domains: (i) methodology, (ii) animal welfare, (iii) reproducibility, and (iv) education.

Methodologically, the platform reduces the potential for unintended bias in experimenter-run tests, which are harder to run blindly. Also, having fully automated tasks reduces the chance of human errors. Moreover, in combination with the automation, the low cost of *GoFish* (Table 1) opens the possibility of testing multiple animals in parallel. Such standardisation across setups and subjects increases efficiency and helps to reduce inter-individual variability, ultimately contributing to a general refinement of procedures.

Methodological refinements will likely result in the reduction of the numbers of experimental animals used. Moreover, eliminating the experimenters´ presence during data collection reduces noises, shadows or other uncontrolled environmental changes thereby reducing subjects’ stress levels, and improving subjects’ welfare.

*GoFish* improves reproducibility, due to standardisation, and highlights the importance of low-cost, open-source tools for the advancement of scientific research. The fact that all components (including software) are open-source should afford further community-based system refinements over the long-term, enabling easier automated extraction of a wider range of behavioural metrics which should enrich the description of behaviour.

It is worth noting that studies have raised concerns regarding the applicability of automated operant training methods for fish, as studies with guppies have shown that automating procedures could lead to slower, unreliable, and task dependent outcomes compared to manually implemented tasks (Gatto et al., 2021). We hope our system, due to its flexibility, would enable us and others to explore this matter further.

The experimental configuration that we present here—a Y-maze setup for two alternative forced choice reversal learning and colour discrimination tasks—is used as a proof-of-concept; *GoFish* is highly adaptable and could be used without configural changes in multiple other experimental paradigms e.g., quantity discrimination experiments (Potrich et al., 2022; Schluessel et al., 2022), behavioural timing (Talton et al., 1999), foraging (Aw et al., 2009; Newport et al., 2021), object recognition (Newport et al., 2016), and navigation (Burt de Perera & Holbrook, 2012). *GoFish* could also be used to implement experiments using a range of set-ups differing to that reported here (e.g., open field and maze configurations that could employ a greater number of screens and/or feeders than we have). It is also worth noticing that *GoFish* could be used to present stimuli, in other sensory modalities: instead of using computer screens for visual stimuli presentation, *Bonsai* affords a large pool of interaction possibilities (e.g., adding a range of sensors and/or actuators, and sound libraries for auditory stimulus generation (Lopes et al., 2021)). Moreover, within *Bonsai*’s framework, our tracking routine, based on colour thresholding, could be extended to implement markerless (Kane et al., 2020 – https://github.com/bonsai-rx/deeplabcut) and multi-animal tracking (Guilbeault et al., 2021; Pereira et al., 2022 – https://github.com/bonsai-rx/sleap). Furthermore, our automatic pellet dispenser could easily be modified to use other regular-shaped rewards by laser cutting a different reward disk (Fig. 1c; see also Arce & Stevens, 2022; Oh et al., 2017). With the present dimensions, the maximum number of rewards between re-fills is 40, which may be limiting for some applications. However, this number depends on the size of individual rewards, which may vary depending on the particular application of the feeder.

Finally, we note that the low price and scalability of the system makes it suitable for hands-on practical experiments and projects in education contexts (e.g., undergraduate projects, summer courses). It could be used for teaching basic animal learning, experimental methods for behavioural research, and data processing (i.e., video tracking) and visualisation.

*GoFish* is a fully integrated, adaptable platform designed to facilitate the implementation of complex behavioural protocols in aquatic species. We hope that our platform accelerates the pace of refined behavioural research in a range of species that otherwise have been relatively underutilised in comparative and cognitive research programmes.

### Supplementary materials

Data File 1 *“Figure3.xlsx”* contains all data presented **in Fig.** **3****.** Each tab corresponds to a panel in the figure. *“panelA”* and *“panelB”* tabs include mean proportion data by session (rows) and animals (columns) for *Experiments* 1 and 2, respectively; *“panelC”* tab contains individual median initiation times (rows) for the first and last sessions' terciles of *Experiment* 2 (columns A:C and D:F, respectively); *“panelD”* tab contains individual median response times (rows) toward stimulus S+ during the first (column A) and last session (column B) of *Experiment* 2, and the corresponding data for responding towards stimulus S- in columns C:D, respectively; *“panelE”* tab contains individual choice data (columns) for the first and last sessions of *Experiment* 2 concatenated vertically (24+24 trials), with ones indicating an S+ choice, and zeros otherwise. To generate Fig. 3, run *"Figure3_forShare.m"* available at: https://github.com/PTMonteiro/GoFish_Ajuwon_etal_2022 (XLSX 13 kb)

Video 1 Example real-time video analysis pipeline for a representative animal and session (AVI 14944 kb)

Video 2 Example of *Bonsai* tracking for a representative animal and session (AVI 27739 kb)

## Declarations

### Data and Code availability

Data is available as a supplementary file (Data file 1) and annotated materials for running the task and data analysis presented in Fig. 3 can be accessed at https://github.com/PTMonteiro/GoFish_Ajuwon_etal_2022.
